# Editorial: Sensorial and perceptual dysfunctions as predisposing factors for the onset of depression

**DOI:** 10.3389/fnins.2022.1094648

**Published:** 2022-11-25

**Authors:** Fabrizio Sanna, Mehmet K. Mahmut, Francesco Loy, Carla Masala

**Affiliations:** ^1^Section of Neuroscience and Clinical Pharmacology, Department of Biomedical Sciences, University of Cagliari, Cagliari, Italy; ^2^Food, Flavour and Fragrance Lab, School of Psychological Sciences, Macquarie University, Sydney, NSW, Australia; ^3^Section of Cytomorphology, Department of Biomedical Sciences, University of Cagliari, Cagliari, Italy; ^4^Section of Physiology, Department of Biomedical Sciences, University of Cagliari, Cagliari, Italy

**Keywords:** depression, olfaction, gustation, vision, somatosensory, earing, proprioception

Olfactory, gustatory, visual, and acoustic disorders are characterized by impairments not only in peripheral stimuli detection (i.e., sensorial level), but also in central processing (i.e., perceptual level). Visual and acoustic impairments affect millions of people around the world, and may have profound impact on quality of life, social communication, psychosocial wellbeing, and economic independence. Visual and acoustic deficits may lead to social isolation, embarrassment, loneliness, and depression.

Similarly, chemosensory dysfunctions (i.e., olfactory and gustatory) may induce comparable limitations on people's quality of life, in particular affecting nutrition and health safety. Recently, the scientific interest in olfactory and gustatory deficits increased due to the impact of the COVID-19 pandemic. In response, this special issue is focused on the description of the neurobiological basis of sensory and perceptual deficits and aims to examine how these processes may be relevant for the onset of depression and mood disorders. In particular, the potential links between chemosensory deficits and the onset of depression were explored given that sensorial disorders may decrease neuronal inputs to limbic circuits which are directly involved in emotional and motivational processes. The Topic presents five articles which range from the animal model to humans in the study of the correlations between different types of sensorial deficits and depression.

The study by Bouguiyoud et al. investigated the associations between vision impairment, depression, and anxiety in a mouse model of congenital blindness. Unexpectedly, the anophthalmic mice did not demonstrate higher depression levels but had lower anxiety associated with an increase in exploratory locomotion compared to the sighted mice. In this context, the use of the animal model allows for an exploration of fundamental mechanisms involved in multi-modal brain plasticity and behavioral adaptations to congenital deficits. In particular, Authors suggested that the behavioral responses in blind mice were associated with brain reorganization and plasticity (for instance, blind mice compensated their absence of visual function by increasing exploratory locomotion of a novel environment).

It is well-known that older humans may exhibit a dual loss of visual and hearing functions, which are associated with decreased quality of life, reduced functional activity, cognitive decline, anxiety, and depression. In this context the study by Killeen et al. examined longitudinal data from the National Health and Aging Trends Study, a representative sample of older United States adults, in the time period from 2011 to 2019 by employing several modeling approaches. The Authors observed that sensory deficits, in particular dual visual and hearing or visual, but not hearing difficulties, were generally associated with a higher risk of depression. Several mechanisms may underlie these associations, among them: social factors such as isolation and loss of social support as well as neurobiological alterations in the function of limbic structures involved in emotion regulation.

The study by Radwan et al. focused on the potential effects of chronic stress on sleep homeostasis in a mice model of susceptibility to stress, namely the C57BL/6J mice, with CD1 mice used as a control. The application of different chronic stress paradigms induced stress-related disorders associated with deficient sleep homeostasis. While stress induced sleep homeostatic processes alterations were evident in both mice lines, a significant impairment in sleep recovery was selectively observed in stress-susceptible mice. These alterations may be associated with an increased risk of developing depression-like symptoms.

Two other articles focused on the association between olfactory deficit(s) and depression in humans. The review by Leon and Woo examined olfactory impairment in the context of at least 68 different neurological disorders in humans, including depression. Based on their extensive analysis of the literature, Authors suggested a potential connection between the olfactory deficit and the neurological alterations. In particular, Authors indicated that olfactory dysfunction may enhance vulnerability to neurological diseases and depression, while olfactory enrichment may decrease such risk.

Finally, Sanna et al. investigated the association between olfactory function and depression in the general population. In particular, Authors observed that the presence of an olfactory impairment was associated with an increased risk of depression, with the sex and an age-related decrease in cognitive functions as intervening factors.

Depression is a psychopathological condition with a multifactorial etiology and with a general trend to increase worldwide. In consideration of the potential negative consequences in those affected by depression, which show a significant burden in quality of life and decreased psychophysical wellbeing, high rates of relapse, and suicide risk, an early detection of risk for depression, potentially with olfactory testing, becomes a key factor to implement effective prevention strategies in the general population.

In conclusion, through an interesting multidisciplinary approach, this Research Topic provides an up-to-date contribution that highlights role of the potential association between sensorial and/or perceptual deficits, as predisposing factors for depression and/or other mood disorders.

## Author contributions

FS, MKM, FL, and CM have made a substantial, direct, and intellectual contribution to the work and approved it for publication.

## Conflict of interest

The authors declare that the research was conducted in the absence of any commercial or financial relationships that could be construed as a potential conflict of interest.

## Publisher's note

All claims expressed in this article are solely those of the authors and do not necessarily represent those of their affiliated organizations, or those of the publisher, the editors and the reviewers. Any product that may be evaluated in this article, or claim that may be made by its manufacturer, is not guaranteed or endorsed by the publisher.

